# Three Adult Cases of Orbital Hidrocystoma Presenting with Blepharoptosis

**DOI:** 10.3390/jcm4010150

**Published:** 2015-01-13

**Authors:** Lucieni B. Ferraz, John R. Burroughs, Larissa H. Satto, Kryscia L. Natsuaki, Roberta L. F. S. Meneguin, Mariangela E. A. Marques, Silvana A. Schellini

**Affiliations:** 1 Ophthalmology Department, Botucatu Medical School, São Paulo Stadual University, São Paulo 18607-370, Brazil; E-Mails: lucienibarbarini@terra.com.br (L.B.F.); larysatto@yahoo.com.br (L.H.S.); kryscial@yahoo.com.br (K.L.N.); rlfsousa@yahoo.com.br (R.L.F.S.M.); sartioli@fmb.unesp.br (S.A.S.); 2 Private Corporation, 111 East Polk Street, Colorado Springs, CO 80907, USA; 3 Pathology Department, Botucatu Medical School, São Paulo Stadual University, Botucatu, São Paulo 18600-010, Brazil; E-Mail: mmarques@fmb.unesp.br

**Keywords:** hidrocystoma, orbital, cyst, apocrine, eccrine, blepharoptosis, orbit

## Abstract

Purpose: To report adult cases of superior orbital apocrine hidrocystoma. Methods: Retrospective case series of three patients with superior orbital apocrine hidrocystoma and blepharoptosis with review of the clinical aspects of each of the cases. Results: All three cases presented with blepharoptosis. Two of the cases had occult hidrocystoma, and one was visibly subcutaneous at presentation. Conclusions: Although rare and more common along the eyelid margin, apocrine hidrocystomas may occur in the orbit leading to secondary blepharoptosis and should be included within the differential diagnosis of orbital cysts. Physicians should therefore be aware of this possibility.

## 1. Introduction

Hidrocystoma cysts derive from sweat glands, and are classified according to the type of secretion as either eccrine or apocrine [1,2,3]. Eccrine hidrocystomas are small, tense thin-walled cysts located mostly at the periorbital and malar areas as either solitary or multiple lesions [4]. Apocrine hidrocystomas, arising from apocrine glands, are common on the head and neck and, when occurring on the eyelids, are usually solitary along the eyelid margin or inner canthus [4].

Their occurrence in the orbit is extremely rare [5,6,7,8,9,10] and only a few cases have been reported, occurring mainly in children [2,7,11] or in adults mostly after trauma [5,12]. We report three unsuspected cases of orbital hidrocystomas that presented with blepharoptosis. The retrospective review of these cases was conducted in compliance with the moral, ethical, and scientific principles governing clinical research as set out in the Declaration of Helsinki (1989).

## 2. Case Reports

**Figure 1:**
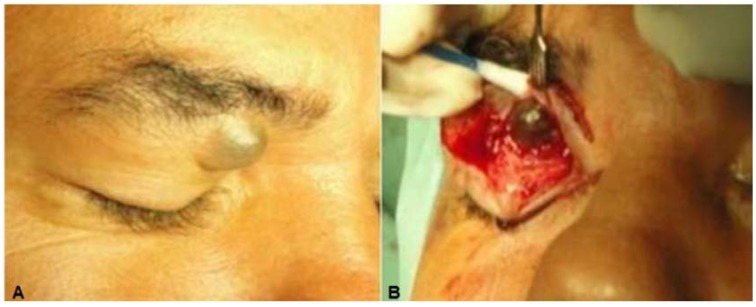

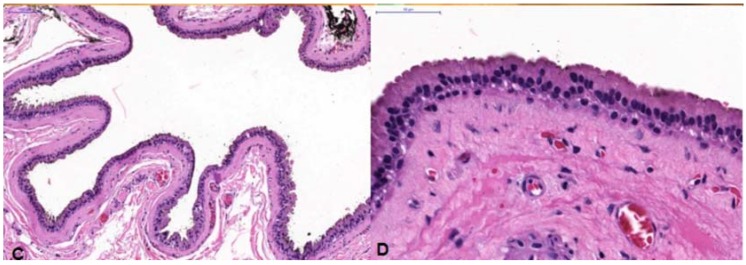
(**A**) Case 1—A bluish round nodule located medially below the right supraorbital margin; (**B**) Cystic lesion above the levator aponeurosis filled by a dark fluid; (**C**) The lesion consisting of a multilocular cyst, lined by a double layer of epithelial cells: an outer layer of flattened vacuolated myoepithelial cells and an inner layer of tall columnar cells with eosinophilic cytoplasm and basally located, round or oval vesicular nuclei (secretory and myoepithelial cells); (**D**) High magnification view of lining epithelium consistent with apocrine hidrocystoma showing decapitation secretion.

**Case 1:** A 46-year-old white man presented a 25-year history of a painless slowly progressive mass in the medial right upper orbital rim. There was no history of previous trauma or ocular disorders. Clinical examination revealed a discrete superior right eyelid ptosis and a nodule located medially below the right supraorbital margin. It was bluish, round, soft, non-tender, vascularized, had no fixation to the overlying skin or the deeper planes, and measured 1.5 × 1.5 cm (Figure 1A,B). Visual acuity, extra-ocular movements, and the ocular exam were otherwise normal. Excisional biopsy of the nodule was performed by anterior orbitotomy with local anesthesia. After opening the orbital septum, a large bluish round cyst was easily removed *in toto*. Histopathological examination showed a multi-loculated solitary cystic lesion surrounded by a single tall columnar cuboidal cell epithelial layer with apical snouting in the lining cells and decapitation secretion (Figure 1C,D), consistent with the diagnosis of apocrine hidrocystoma.

**Figure 2:**
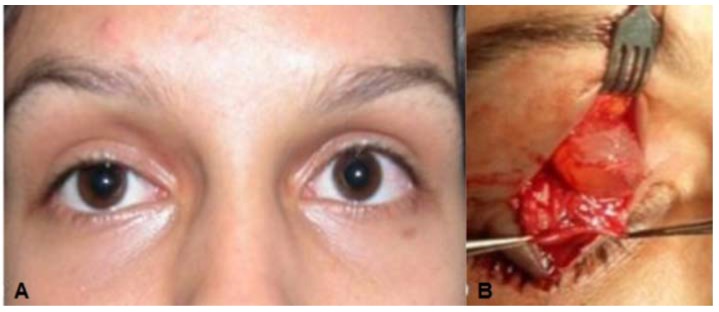

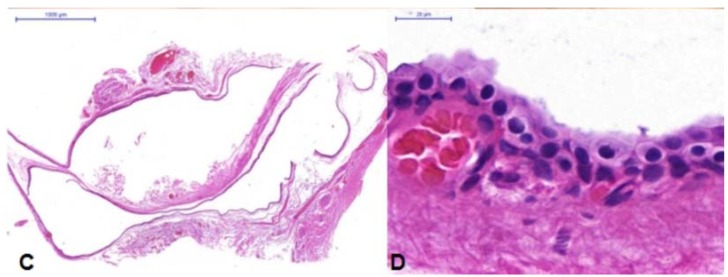
(**A**) Case 2—Ptosis of the right upper eyelid; (**B**) Cystic lesion with clear liquid content among the fibers of the upper eyelid levator muscle; (**C**) Double layered cuboidal cell lining, areas of transitional epithelium and positive apical glycocalyx with apical snouting; (**D**) High power view of lining epithelium consistent with apocrine hidrocystoma showing decapitation secretion.

**Case 2:** A 17-year-old white woman complaining of congenital right upper eyelid ptosis (Figure 2A) with no change since birth. She was born by vaginal-delivery without complications, and there were no other previous ocular disorders. The visual acuity, extra-ocular movements, and ocular exam were otherwise normal. A diagnosis of mild congenital blepharoptosis was made and the patient wished to have surgical correction. During surgery, after opening the orbital septum, a round cyst measuring 1 × 1 cm containing clear liquid was observed rising among the fibers of the levator muscle (Figure 2B). This solitary cystic lesion, without any adherence to the surrounding tissues, was easily removed *in toto*. Histological examination revealed the cystic lesion was lined by simple columnar epithelium and decapitation secretion is seen consistent with a diagnosis of apocrine hidrocystoma (Figure 2C,D).

**Figure 3:**
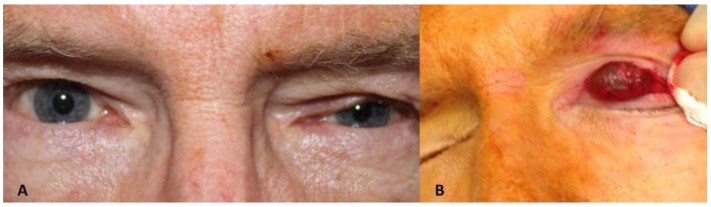

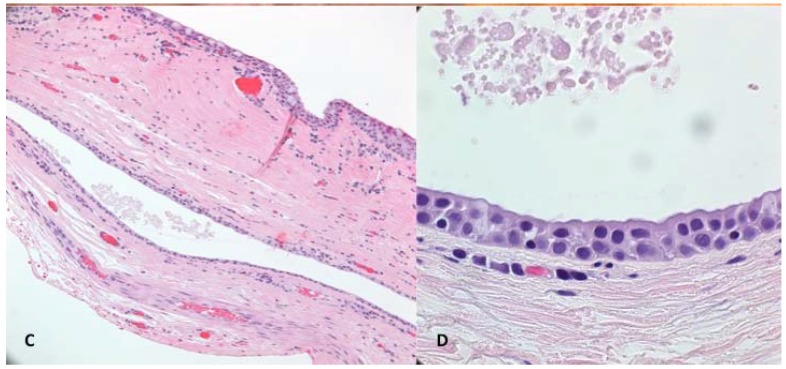
(**A**) Case 3—Ptosis of the left upper eyelid; (**B**) Apocrine Cystic lesion with bluish color; (**C**) Low magnification photomicrograph. Superior portion of the specimen shows conjunctival tissue with goblet cells that had been excised with cyst. The middle portion shows the cystic lining; (**D**) High magnification of the cyst lining showing multi-layered cuboidal epithelium with the luminal surface showing faint apocrine secretions most consistent with apocrine hidrocystoma.

**Case 3:** A 67-year-old man, who had been a long-standing hard contact lens wearer, seemingly “lost” his left contact lens. He never recalled removing it, and about 10 years later it spontaneously reappeared on his eye. Approximately 15 years after the contact lens reappeared, he presented for a left-sided blepharoptosis repair. He had an unusual presentation, with the good levator function ptosis affecting mostly the nasal half of his upper eyelid (Figure 3A). The preoperative ocular exam was otherwise unremarkable, and he had no complaints of a mass sensation in his upper eyelid and no abnormalities were palpated on exam. During the external levator ptosis surgery, upon opening the septum a large (1.9 × 1.6 × 1 cm) bluish-red cystic mass prolapsed forward (Figure 3B). It was adherent to the levator aponeurosis but otherwise easily resected. Wide resection was performed to remove the cyst intact, so a portion of conjunctiva was included in the specimen. The levator ptosis repair was then completed in standard fashion. Histology of the mass (Figure 3C,D) was most consistent with an apocrine hidrocystoma as it was a singular cystic lesion of large size with dual-layered epithelial cells and the absence of goblet cells in the cyst wall. Some goblet cells were seen in the specimen where portions of conjunctiva were present.

## 3. Discussion

Congenital sudoriferous cysts are related to embryonic epithelial cells, destined to form glands of Moll, becoming sequestered leading to cyst formation in the orbit [13] and because of this orbital hidrocystomas occur mainly in children [2], although our three cases were all adult at time of presentation. The first case was self-detected by the patient in their 20’s while the second patient had mild blepharoptosis since childhood, which may suggest the cyst was there since birth. Neither of these first two cases had any history of prior trauma, so, we presume they were of congenital origin and grew gradually, as has been previously reported [13].

The third patient was elderly at the time of discovery during an external levator ptosis surgical repair. Apocrine cysts arising in adults are commonly related to traumatic implantation of epithelial cells into deeper tissues leading to formation of secondary epithelial inclusion cysts [12]. It is possible that the hard contact lens that had “gone missing” caused traumatic disruption to the superior fornix leading to the formation of an occult hidrocystoma that only became discoverable at the time of the blepharoptosis surgery. Because conjunctival features within the cyst portion of the specimen were not seen histologically, the diagnosis of a traumatic conjunctival implantation cyst is possible but less likely.

Blepharoptosis was present in all three of our patients with only one of the cases having a known painless cystic mass prior to surgery. These patients did not otherwise have ocular complaints, but reported symptoms for periocular hidrocystomas include: eyelid swelling; eye pain; headaches; visual discomfort; strabismus; exophthalmia; and visual field defects [5,11,14].

The first case’s clinical exam showed a non-tender, non-adherent, and well-delineated lesion. The lesions were not detected in the other two cases before ptosis repair surgery was undertaken. Despite the orbital location in each of our patients, gross exophthalmos was not present. This could be explained by the anterior location of the cysts and/or the possible presence of bony remodeling following their slow growth along the superior orbital rim. Computed tomography or magnetic resonance imaging can help identify these lesions, but are not necessarily helpful in narrowing the differential diagnosis [5,7].

The clinical appearance and location may suggest the derivation of hidrocystomas. Apocrine hidrocystoma are found at the eyelid margin in association with the strong cilia and caruncle area, anogenital region, axillary, areolar; they are generally larger (up to 20 mm). Eccrine hidrocystoma, in contrast, are usually small (1–3 mm), located exclusively in the periorbital and lateral canthal area and they are absent at either the upper or lower eyelid margins or in the perimarginal skin, but may be rarely present in the dermis typically at the mid-level of the upper eyelid pretarsal skin, or in the lower eyelid skin near the inferior pole of the lower eyelid tarsus [15].

Sudoriferous cysts arising from the sweat glands of Moll are usually found on the eyelids and have apocrine origin [12]; they are easily recognized clinically because of their typical appearance as a translucent, clear fluid-filled cyst with a smooth surface [5]. However, fluid color may vary, ranging from flesh-colored to a blue-black because of the presence of lipofuscin, the Tyndall effect, and if melanocytes are present [5]. The bluish coloration observed in two of our three patients is an unusual appearance and may also be seen with: hemangioma; dermoid or teratogenous cyst; hydatic cyst; neural cyst; inflammatory cyst; secondary cyst from adjacent structures (skin or conjunctival); and malignant melanoma. Acquired orbital cysts also include mucoceles, conjunctival implantation cysts, and lacrimal ductal cysts [4,16,17].

Hidrocystomas are microscopically distinguished based on the production of secretion in two varieties: (1) apocrine in which the lining cells release the apical part of their cytoplasm and the remainder of the cell being viable; and (2) eccrine, which sustain no loss of cell structure [12]. Apocrine hidrocystomas staining by hematoxylin and eosin are seen as a unilocular or multilocular cyst, with the cyst wall composed of an inner layer of secretory columnar epithelium and a single or double layer of cuboidal-columnar epithelium, overlying an outer myoepithelial cell layer that has papillary projections not usually seen in the eccrine hidrocystomas [5,17]. Typical apocrine features of hidrocystomas have been described as a cystic structure with dual layered epithelial cells [2,6] and exhibiting a deeply eosinophilic cuboidal to columnar cellular lining with oval to round basal nuclei and apical cytoplasmic decapitations (shedding of adlumenal snouts incorporating into the eosinophilic secretions along with specific granules) have conventionally been designated as apocrine [15,18]. In contrast, cysts lined by a low cuboidal or flattened epithelium with paler cytoplasm, no snouts and a watery secretion without cytoplasmic contributions have been classified as eccrine hidrocystomas. The presence of decapitation secretion and myoepithelial cells in apocrine hidrocystoma are a useful distinguisher from eccrine [18]. Another important observation is that hidrocystomas contain no goblet cells, which distinguishing them from conjunctival cysts [19].

There are more sophisticated tests to distinguish apocrine from eccrine hidrocystoma in the unaided light microscopy cases: human milk fat globulin-1 (1.10.F3) monoclonal antibody, cytoplasmic granules containing epidermal growth factor, gross cystic disease fluid protein-15 (GCDFP-15), cytokeratin 7 (CK7), alpha-smooth muscle actin (αSMA) [12,15,16] and others which are not universally available or clinically critical.

Histological findings also enable hidrocystomas to be distinguished from other lesions such as: cystadenoma, which will show a cystic lesion with an adenomatous component; dermoid cysts characterized by keratinizing squamous epithelium with a keratohyalin layer and fibrous wall that may contain adnexal structures (e.g., hair) [15,16,17]; conjunctival dermoids usually contain mucous-containing goblet cells sometimes with a partially keratinized lining; simple conjunctival cyst showing nonkeratinizing squamous epithelium or a double row of low cuboidal, non-ciliated cells or flattened squamous cells with admixed goblet cells [19]; dacryocystocele/dacryomucocoeles lined by pseudostratified columnar cells with scattered goblet cells; canaliculops shows a dilated canaliculus lined by squamous cells multiple layers thick and enveloped by striated orbicularis muscle fibers; dacryops lined by a double layer with cuboidal and columnar cells with scattered goblet cells sometimes present; respiratory enteric cysts, which show a lining of ciliated or not ciliated columnar epithelium with scattered goblet cells; and cystic odontogenic choristomas, which show variable cyst linings, multiple potential other glands (e.g., mucous), and one or two teeth [15,16,17,20].

A failure to evaluate the cytologic subtleties may lead to misdiagnosing hidrocystoma. The most common error is to mislabel a cyst as a hidrocystoma instead of conjunctival cyst [20]. A true cystadenoma has a composite nature wherein the mass has a mostly cystic component with a solid adenomatous (gland-forming) component. Unfortunately the term of a cystadenoma has been ascribed to apocrine cysts and hidrocystomas and as distinct from eccrine hidrocystomas. This practice is not optimal unless the composite nature as just described is noted histologically [20]. 

Though surgical excision is not generally difficult, incomplete excision of orbital cysts carry high risk for recurrence [21]. Eyelid specific treatments for hidrocystomas have included carbon dioxide vaporization, electrosurgery, hyfrecation, botulinum toxin, and intralesional tricholoroacetic acid injection [15,22,23].

Fortunately in our three cases the diagnosis was benign, but this may not always be the case and some orbital lesions, if not fully excised, carry malignant transformation potential. As highlighted in this case series, unusual blepharoptosis patients should be referred to an oculoplastic-experienced surgeon with orbital experience to ensure optimal patient safety and outcome.

## 4. Conclusions

In conclusion, although rare and most common along the eyelid margin, hidrocystoma should be included in the differential of orbital cysts. It is important to be aware of the variable presentation of orbital hidrocystomas. Unusual blepharoptosis, dysmotility, globe displacement, and/or proptosis are warning signs of a potential underlying secondary cause, such as an orbital mass. Patients presenting with unusual blepharoptosis (e.g., unilateral; asymmetric) should undergo upper eyelid palpation, eyelid eversion, a thorough ocular exam, extraocular movement testing, be considered for orbital imaging and a surgical approach that will help exclude an anterior orbital mass as the cause.
